# Lung cancer brain metastasis and hemorrhagic cerebral venous thrombosis: experiences and lessons

**DOI:** 10.1186/s12959-024-00629-0

**Published:** 2024-07-11

**Authors:** Qilong Tian, Yingxi Wu, Gang Li, Xiaofeng Huang, Qing Cai

**Affiliations:** 1 Department of Neurosurgery, Tangdu hospital, Air Force Medical University, Xi’an, China; 2 Department of Oncology, Tangdu hospital, Air Force Medical University, Xi’an, China

## Abstract

The incidence of lung cancer brain metastasis combined with hemorrhagic cerebral venous sinus thrombosis (CVST) is very rare, and the understanding and treatment experience of this case is insufficient. We reported a case of lung cancer brain metastasis accompanied by venous sinus thrombosis, and describe the diagnosis and treatment plan for colleagues to learn from experience and lessons.

## Key points

**1. ** The incidence of lung cancer brain metastasis combined with hemorrhagic CVST is very rare, and the understanding and treatment experience of this case is insufficient.

**2. ** The clinical manifestations of metastatic tumors and CVST in patients are not specific, and the evolution of the medical history should be inquired in detail, with careful physical examination and imaging review.

**3. ** CVST should be considered in patients with risk factors. The incidence of hemorrhagic CVST is relatively rare, and due to atypical symptoms and imaging manifestations, it is easy to miss and misdiagnose.

**4. ** The responsible lesion should be determined based on the evolution of the medical history, the treatment of the responsible lesion should be prioritized, symptoms should be relieved in a timely manner and the optimal treatment time should be strived for.

A 49-year-old female patient was admitted to a hospital after undergoing lung cancer treatment for 2 years and having complaints of headaches with unsteady walking for 1 month. The patient visited the hospital 2 years previously due to progressive enlargement of a neck mass for more than half a month. Chest CT showed a nodular shadow in the upper lobe of the right lung with peripheral inflammation, multiple lymph node shadows in the mediastinum. The pathological diagnosis by pulmonary nodule puncture biopsy was adenocarcinoma grade II. Further genetic testing revealed EGFR exon 19: c.2235_2249del: p.745_750del. The patient was administered gefitinib tablets and underwent regular examinations. PET-CT (9 months ago) showed that there was a slight increase in glucose metabolism in the primary lung lesion and mediastinal lymph nodes. Despite the changes in metabolic activity after tumor treatment, there was no significant increase or hypermetabolic lymph node shadow on either side of the neck. No mass or abnormal glucose metabolism changes were observed in the brain or trunk.

**Fig. 1:**
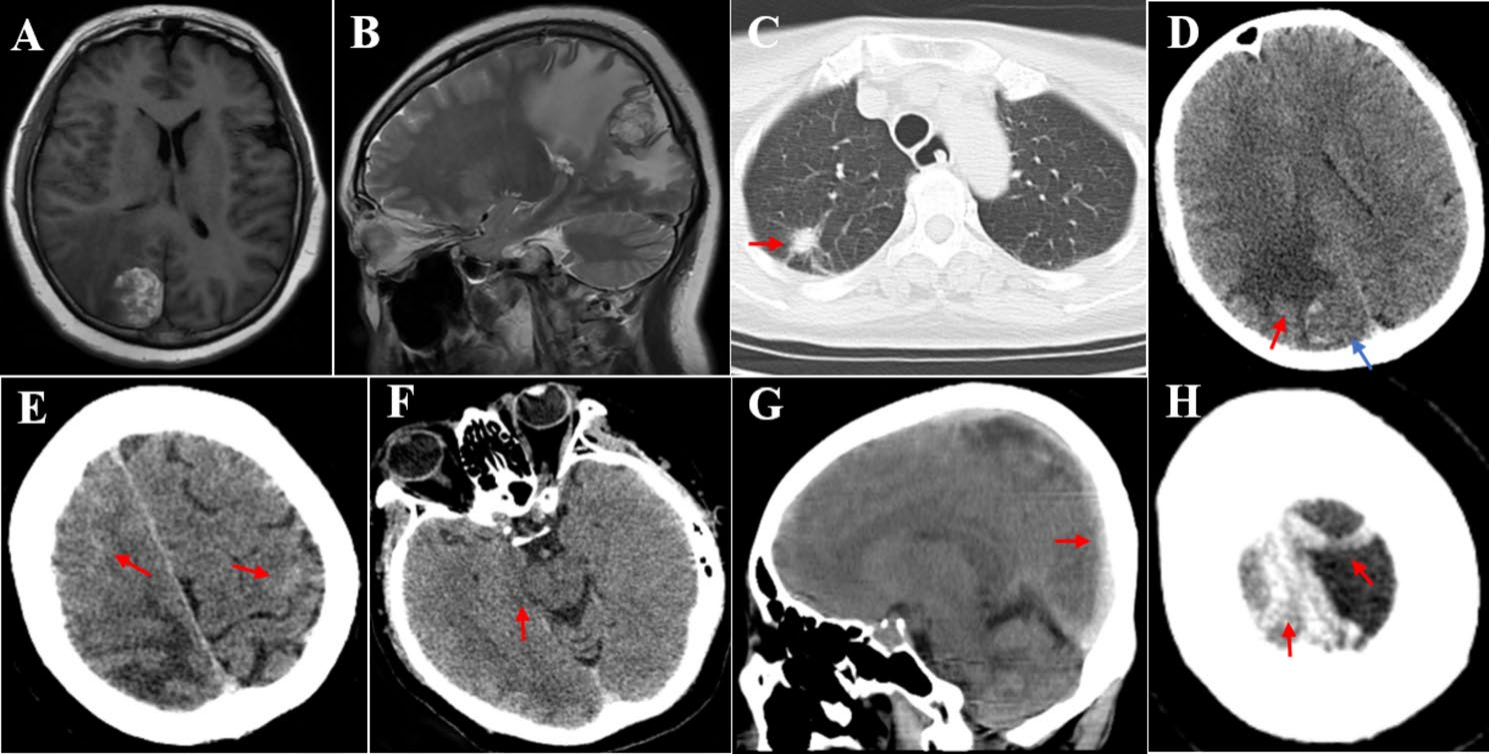
preoperative intracranial metastasis and CVST with SAH. (**A**) Metastasis in the right parietal-occipital lobe. (**B**)Severe peritumoral edema. (**C**)Right upper lobe adenocarcinoma (red arrow). (**D**) Metastasis (blue arrow) and increased peritumoral edema (red arrow). (**E**) Minor SAH in bilateral parietal lobes (red arrow). (**F**) Compression of right cisterna (red arrow). (**G**) Hyperdense SSS (red arrow). (**H**) Hyperdense cortical vein (red arrow)

**Fig. 2:**
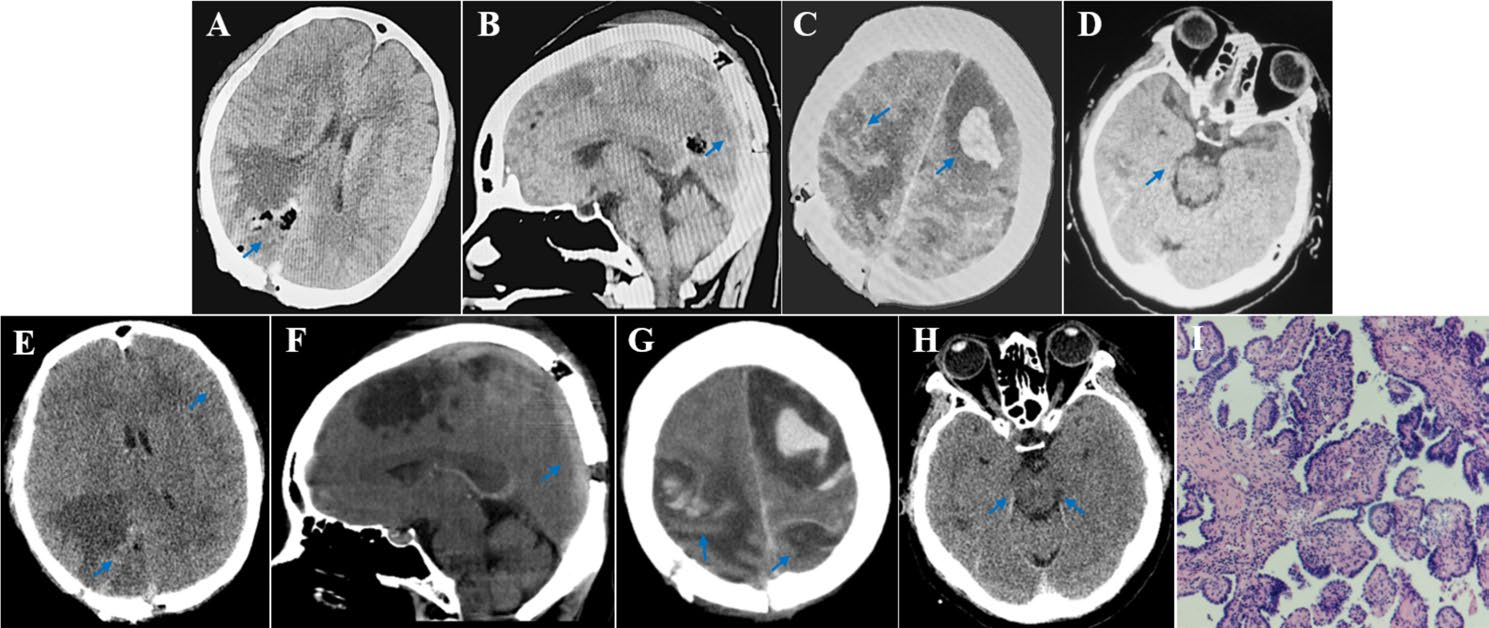
Postoperative metastasis resection and intracranial hemorrhage in CVST. **A-D**: CT display at postoperative 10 h. (**A**) Complete metastasis resection (blue arrow). (**B**) Hyperdense SSS (blue arrow). (**C**) added left frontal lobe hematoma and right parietal lobe edema with minor bleeding (blue arrow). (**D**) Compared to preoperation, the compression of the right cistern was relieved (blue arrow). **E-H**: CT display at postoperative 30 h. (**E**) Disappearance of tumor cavity and sulcus, diffuse brain swelling (blue arrow). (**F**) Hyperdense SSS (blue arrow); (**G**) Increased bleeding in the right parietal lobe and significant edema in the left parietal lobe (blue arrow). (**H**) Obvious compression of bilateral cisterns (blue arrow). (**I**) Pathological lung adenocarcinoma with brain metastasis

The patient experienced worsening of the progressive headaches, nausea and vomiting, and unsteady walking that had begun 1 month prior. Head MRI displayed a mass lesion in the right parietal-occipital lobe with obvious peritumoral edema, suggesting brain metastasis from lung cancer (Fig. 1A and B). A chest CT scan showed no enlargement of the lesion in the upper lobe of the right lung (Fig. 1C). The patient was treated with mannitol and methylprednisolone after admission. The patient’s consciousness turned to drowsiness, with a right pupil size of 4 mm and dull light reflex and left limb hemiplegia (left upper limb muscle strength level 1 and lower limb level 2). Head CT demonstrated obvious peritumoral edema (Fig. 1D), minor subarachnoid hemorrhage (SAH) in the bilateral parietal lobes (Fig. 1E), and high-density shadows in the superior sagittal sinus (SSS) (Fig. 1G) and cortical veins, suggesting cerebral venous sinus thrombosis (CVST) involvement of cortical vein thrombosis with hemorrhage (Fig. 1H). After further deterioration of the patient’s awareness, right parietal occipital metastasis resection was performed under general anesthesia. The patient was conscious 2 h postoperatively with a 2 mm right pupil size and sensitive light reflex. The left limb muscle strength improved compared to before (left upper limb muscle strength level 3, lower limb level 4). Unfortunately, regarding the patient’s consciousness, the patient became drowsy and lethargic at 6 h and 10 h postoperatively, respectively. Head CT revealed that the metastasis in the surgical area was completely removed (Fig. 2A), with newly developed left frontal lobe bleeding and edema (Fig. 2C), which were considered to be from CVST accompanied by cortical venous hemorrhage. Her consciousness slightly improved after strengthening dehydration and fluid management. However, the patient’s consciousness deepened to a coma 30 h postoperatively with a left pupil size of 4 mm and dull light reflex. CT indicated disappearance of the sulcus and gyrus, diffuse swelling of supratentorial brain tissue, and formation of a cerebral hernia (Fig. 2E-H). Bilateral bone flap decompression was recommended to relieve her intracranial pressure, but surgery was not performed. The patient died 72 h after surgery.

## Discussion

This is a rare case of lung cancer brain metastasis combined with CVST and involvement of cortical vein thrombosis associated with hemorrhage. The incidence of brain metastasis from lung cancer is as high as 50% [1]. A single metastasis with an obvious mass effect and increased intracranial pressure can be surgically removed. The metastasis was located in the right parietal-occipital lobe, with severe peritumoral edema and a significant mass effect, indicating that there were surgical indications [2]. The incidence of cerebral venous thrombosis accounts for 0.5-1% of all adult stroke patients, and CVT is associated with intracranial hemorrhage in up to 40% of patients [3, 4]. Imaging of CVT on CT include indirect signs (edema, parenchymal hemorrhage, SAH, and rarely subdural hematomas) and less commonly direct signs (visualization of dense thrombus within a vein or within the cerebral venous sinuses). Confirmation is performed with CTV, directly demonstrating the thrombus as a filling defect (CTV/MRV) [5, 6]. Venous sinus thrombosis is more common in young women, especially those taking oral contraceptives, pregnant, and postpartum. This also includes risk factors for venous thrombosis, such as hereditary thrombotic, inflammatory diseases and cancer. Situations that particularly increase the risk of CVT include head trauma, arteriovenous malformations, neurosurgery, and head and neck infections [7]. Overall, approximately 85% of patients can detect related diseases [8]. First-line treatment for CVT is heparin, even in the presence of an intracerebral Haemorrhage. Endovascular treatment should not be routinely applied in patients with CVT because it did not improve the clinical outcome of patients with severe CVT. Decompressive craniectomy is recommended as an emergency treatment for cerebral herniation and can achieve a good prognosis [7, 9, 10].

The patient had multiple risk factors for CVST, including age (20–50 years), sex (female), systemic diseases (cancer), and uncertain adverse reactions (gefitinib tablets). CT showed direct signs (hyperdense SSS and cortical vein) and indirect signs (edema, parenchymal hemorrhage, SAH). Based on clinical manifestations and imaging examinations, metastasis and CVST require treatment. Whether prioritizing the treatment of metastasis or CVST, there may be extreme complications. Tumor resection may induce aggravation of CVST. When choosing anticoagulation or interventional therapy for CVST, metastasis and peritumoral edema cannot be eliminated or can even worsen. Simultaneously, there was short-term loss of surgical opportunity (prone to bleeding). Detailed analysis of the patient’s condition development revealed that the patient had high intracranial pressure and corresponding neurological dysfunction preoperatively (changes in the right pupil, hemiplegia in the left limb), accompanied by suspected CVST and SAH. Both may contribute to deepening consciousness, but metastasis and peritumoral edema are primarily responsible for pupil changes and limb disorders.

We chose to first remove the tumor to alleviate the mass effect of the metastasis and peritumoral edema. The patient’s postoperative consciousness and pupil and limb function improved, which also confirmed our preoperative judgment. If postoperative CVST does not worsen, anticoagulation or interventional treatment could be considered. Unfortunately, postoperative edema and bleeding of cortical veins occurred one after another, leading to a sharp increase in the intracranial pressure and the formation of a cerebral hernia. Conversely, choosing thrombolytic therapy first is also highly likely to lead to multiple bleeding in the brain parenchyma, plus metastasis and peritumoral edema mass effects, with dual effects ultimately leading to a poor prognosis. Therefore, the patient’s metastasis combined with hemorrhagic CVST means that the patient was at the end stage of the disease, which requires active intervention in a timely manner. However, the treatment is contradictory, and any method may result in adverse outcomes.

## Data Availability

No datasets were generated or analysed during the current study.

## Abbreviations

CVST: Cerebral venous sinus thrombosis
SAH: Subarachnoid hemorrhage
SSS: Superior sagittal sinus
